# Clinical Characteristics and Risk Factors of Periprocedural Myocardial Injury in Patients with Elective PCI in a TCM Hospital

**DOI:** 10.1155/2022/7158740

**Published:** 2022-03-24

**Authors:** Xiang Li, He-Yi Zhang, Ju-Ju Shang, Hong-Xu Liu, Qi Zhou, Xiao-Lei Lai

**Affiliations:** ^1^Department of Cardiology, Beijing Hospital of Traditional Chinese Medicine, Capital Medical University, Beijing 100010, China; ^2^Clinical Medical School, Beijing University of Traditional Chinese Medicine, Beijing, 100029, China

## Abstract

**Objective:**

To investigate the clinical characteristics of patients with unstable angina (UA) who received elective percutaneous coronary intervention (PCI) in a traditional Chinese medicine (TCM) hospital and to analyze the related risk factors of periprocedural myocardial injury (PMI).

**Methods:**

On the basis of cross-sectional investigation, the case-control method was adopted. We retrospectively collected clinical data of patients with UA who successfully received elective PCI in Beijing Hospital of TCM from February 2017 to February 2019. Based on the occurrence of PMI, the case-control was formed. The influence of related factors on PMI occurrence was analyzed using the logistic multiple regression equation based on the parameters between the comparison groups.

**Results:**

1. Incidence of PMI and related clinical features: Of the 265 UA patients, the incidence of PMI was 26.4%, nearly one quarter (23.4%) had old myocardial infarction, nearly half (45.3%) had previously received coronary intervention. The prevalence of patients with previous hypertension (75.8%), type 2 diabetes (57%), and high-low-density lipoprotein cholesterolemia (69.3%) exceeded 50%, more than 50% of the patients have triple-vessel disease (50.2%). 2. Features of TCM syndrome elements: The main TCM syndromes of the investigated patients are blood stasis syndrome (81.1%) and Qi deficiency syndrome (77.3%), the others include Phlegm turbidity syndrome (53.2%), Yang deficiency syndrome (50.9%), Yin deficiency syndrome (50.1%), Qi stagnation syndrome (30.1%), and coagulated cold syndrome (17.1%). 3. Factors of PMI occurrence: According to the occurrence of PMI, 265 patients were divided into PMI group (*n* = 70) and non-PMI group (*n* = 195). The comparison between groups shows that the preoperative SYNTAX score, the number of stents, and the total length of stents of the patients in the PMI group were higher than those in the non-PMI group (*P* < 0.05); the patients in the PMI group treated by Shen-Yuan-Dan (SYD), a Chinese medicine prescription with Qi-supplementing and blood stasis-purging, were significantly lower than those in the non-PMI group (*P* < 0.05). Brought these four factors (preoperative SYNTAX score, number of stents implanted, total length of implanted stents, and treated by SYD) into the binary logistic regression equation, those who were only treated by SYD have statistical significance in the equation as a protective factor (OR 0.327, 95% CI 0.117–0.916, *P*=0.033).

**Conclusion:**

Patients with UA who received elective PCI in TCM institutions may have clinical characteristics including multiple accompanying diseases and high stenosis coronary artery, in which the incidence of poor blood glucose control and high rate of three-vessel coronary disease are particularly significant. The TCM syndromes are mainly Qi deficiency and blood stasis syndromes. The decrease of PMI may be attributed to the application of SYD in the real world. This trial is registered at ChiCTR2100043465.

## 1. Introduction

Periprocedural myocardial injury (PMI) is a common factor that hinders the clinical benefit of patients receiving percutaneous coronary intervention (PCI) [1], and PCI has been widely carried out in traditional Chinese medicine (TCM) institutions in China. Research studies [2] showed that the clinical characteristics of patients with acute myocardial infarction (AMI) treated in hospitals of TCM were different from those in hospitals of western medicine, with the unique clinical characteristics such as high age of onset and more associated diseases, which suggested that patients undergoing PCI and patients occurring PMI in TCM hospitals might have independent clinical features. However, there are few reports on those mentioned above. Therefore, the purpose of this study was to investigate the clinical characteristics of patients with unstable angina (UA) who received selective PCI in a TCM hospital and to analyze the related risk factors for the occurrence of PMI so as to provide ideas for the prevention and treatment of PMI in TCM institutions which are special and numerous.

## 2. Materials and Methods

### 2.1. Data Source of Patients

The data used in this study were obtained based on the Electronic Medical Records (EMRs) in Beijing hospital of TCM from February 2017 to February 2019.

### 2.2. Diagnostic Criteria

#### 2.2.1. Diagnostic Criteria of PMI and PMIf

Patients with normal cardiac biomarkers (cTnI) are diagnosed with PMI if the postoperative biomarkers' increase exceeds the 99th percentile of the upper limit of normal. Periprocedural myocardial infarction (PMIf) will be diagnosed if the postoperative cardiac biomarkers' increase exceeds 5 times more than the 99th percentile of the upper limit of normal, and there are symptoms of cardiac ischemia or coronary angiography and other imaging evidence [3].

#### 2.2.2. Diagnostic Criteria of TCM Syndrome

The TCM syndrome element and type diagnosis standard were as follows: the Syndrome Part of TCM Clinical Diagnosis and Treatment Terminology (the National Standard of the Peoples' Republic of China) [4], TCM Diagnostics [5], and Syndrome Elements Differentiation [6].

1. *Qi Deficiency Syndrome.* Chest pain or palpitations, accompanied by shortness of breath, listlessness, spontaneous sweating, pallor, pale tongue‚ and weak or irregular pulse.
2. *Yin Deficiency Syndrome.* Chest pain or palpitations, accompanied by mental irritability, palpitation, insomnia, low fever, night sweating, malar flush, thirst, and rapid fine pulse.
3. *Yang Deficiency Syndrome.* Chest pain or palpitations, accompanied by reversal cold of limbs, feeble breathing, clouding or loss of consciousness, pale complexion, and hardly perceptible pulse.
4. *Blood Stasis Syndrome.* Chest stabbing pain, palpitations, purple tongue or tongue with purple spots, and fine choppy pulse.
5. *Qi Stagnation Syndrome.* Chest pain or palpitations, accompanied by depressed mood, frequent sighing, borborygmi with flatus and white slimy tongue coating, and string pulse.
6. *Coagulated Cold Syndrome.* Chest pain or palpitations, mostly due to sudden cold climate, accompanied by cold shape, shortness of breath, pale face, light tongue with white moss, and tight pulse.

### 2.3. Inclusion Criteria

According to the *International Classification of Diseases* (ICD) from EMRs in Beijing hospital of TCM, in-patients whose first diagnosis was UA and who successfully received elective PCI were included.

### 2.4. Exclusion Criteria

Patients with incomplete medical records, prescription medication data, and other information were excluded.

### 2.5. Data Collection Content

(1) General information about the patients: demographic profile: name, gender, and age; risk factors: smoking and alcohol consumption; previous medical history: hypertension, type 2 diabetes, high LDL-C, cerebrovascular disease, old myocardial infarction, previous PCI, and previous CABG; and first biochemical parameters on admission: lipids, blood glucose, cardiac, liver and renal function, and glycosylated haemoglobin. (2) Occurrences of PMI. (3) Features of the patient's TCM evidence on first admission. (4) Information about PCI includes nature of lesion, site of lesion, preoperative GRACE score, and preoperative SYNTAX score in patients undergoing coronary interventions; coronary interventions are the number of stents implanted, total length of stents implanted, and the total time of dilation. (5) Guideline recommendations for secondary prevention of coronary heart disease: antiplatelet agents, beta-blockers, statins, and angiotensin-converting enzyme inhibitors (ACEI)/angiotensin receptor blockers (ARB) drugs. (6) Application of TCM treatment: involving information of TCM intravenous preparations and Chinese patent medicine or Chinese herbal medicine application for at least 3 days before PCI. (7) Application of prescription (Shen-Yuan-Dan (SYD)) with function of Qi-supplementing and blood stasis-purging: a capsule preparation of the SYD or a Chinese herbal tablet preparation containing the core components of the SYD applied before PCI (judging criteria: a formula embodying the treatment principles of Qi-supplementing and blood stasis-purging and including all 8 herbal medicines in the SYD and applied for at least 3 days, evaluated by 2 physicians with senior titles at the same time).

### 2.6. Data Entry and Management

The case survey data for this study were set up on a data platform through an EXCEL software base. Data from the questionnaires were entered by trained entry clerks separately and uniformly for 2 times, with error checking through professional software after the 2 entries. All observations and findings relating to the study should be verified, and quality control must be carried out at each stage of data processing to ensure that the data are complete, accurate, authentic, and reliable. This project involves a dedicated person for cases that need to be collected.

### 2.7. Observed Indicators

The clinical characteristics of UA patients undergoing elective PCI in Beijing hospital of traditional Chinese medicine include basic patient information: age, gender, history of smoking and drinking, underlying disease, lesion site, lesion nature, preoperative Grace score, preoperative SYNTAX score, etc.; information on Chinese medicine evidence; coronary interventions: the number of stents implanted, total length of stents implanted, average diameter of stents, and total time of dilation; application of western medicine: antiplatelet, lipid-lowering, *β*-blockers, etc.; application of TCM treatment (involving all application information of TCM intravenous preparations, Chinese patent medicines, or Chinese herbal medicines); application of TCM intravenous preparations (involving application information of Danhong injection and Tanshinone IIA injection); application of Chinese patent medicines (involving application information of compound Danshen dripping pill and Tongxinluo capsule); and application of Chinese herbal medicines and the application of SYD (no less than 3 days).

### 2.8. Assessment of Sample Size

We evaluated the sample size according to the following sample size calculation formula of the cross-sectional study [7]:

(1) $$\begin{array}{c} n=\frac{U_{1-\alpha /2}^{2}P_{0}\left(1-P_{0}\right)}{d^{2}}, \end{array}$$

where *P*_0_ represents the expected occurrence of PMI in this study. According to the results of related studies, the incidence of PMI ranged from 4.7 to 40.5% [1, 8–10]. We assumed that *P*_0_ was 30%. In this formula, *U*_1−*α*/2_ = 1.96, *d* represents *survey error*. We assumed *d* was 0.2 times of *P*_0_. Therefore, a total of 224 patients should be needed.

### 2.9. Statistical Methods

Statistical software SPSS software version 27.0 (IBM Inc., New York, USA; Account Name: Beijing Hospital of TCM, CCUM) was used for statistical analysis. The measurement data were expressed as mean ± standard deviation. Comparisons between groups of multiple normally distributed continuous variables with homogeneous variances were performed by analysis of variance with a completely randomized design (One-way ANOVA), and two-way comparisons between multiple groups were performed by the SNK-*q* test. Comparisons between groups of nonnormally distributed continuous variables were made using the rank sum test. The chi-square test was used for intergroup comparisons of count data, and the Fisher exact probability test was used when the expected frequency was <1. Binary logistic regression analysis was performed with the occurrence or nonoccurrence of PMI as the dependent variable (occurrence of PMI = 1 and nonoccurrence of PMI = 0), and factors were statistically different between group comparisons as independent variables, using the forward LR method, with a test level of 0.1 and a rejection level of 0.15 for the independent variables entering the regression model. *P* < 0.05 was considered a statistically significant difference.

## 3. Results

### 3.1. Clinical Features and Occurrence of PMI

Two hundred and sixty-five patients with UA who successfully underwent elective coronary intervention were selected from February 2017 to February 2019 at Beijing hospital of TCM. Of the 265 patients, 70 patients (26.4%) experienced periprocedural myocardial injury (PMI) and 12 patients (4.5%) experienced periprocedural myocardial infarction (PMIf) with a high level of postoperative cardiac biomarkers that exceed 5 times more than the 99th percentile of the upper limit of normal, but none of these 12 patients were re-examined by coronary angiography after PMIf diagnosis. The 265 patients investigated were divided into PMI group (*n* = 70) and non-PMI group (*n* = 195) according to whether they had PMI or not. The general clinical data of the patients were compared and analyzed (Table 1).

**Figure 1:**
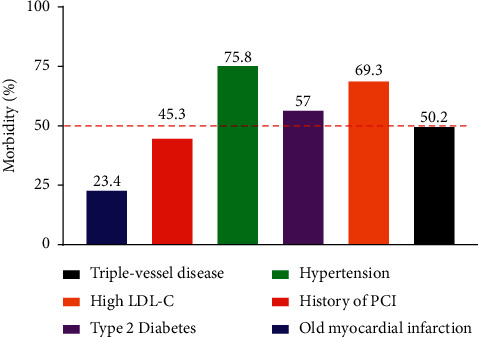
Clinical characteristics of patients underwent PCI.

Of the 265 patients surveyed who underwent coronary intervention, nearly one quarter (23.4%) had old myocardial infarction; nearly half (45.3%) had previous coronary intervention; the prevalence of patients with previous hypertension (75.8%), type 2 diabetes (57%), and high LDL-C (69.3%) was all over 50%; and over 50% of the patients have triple-vessel disease (50.2%) (Figure 1).

**Figure 2:**
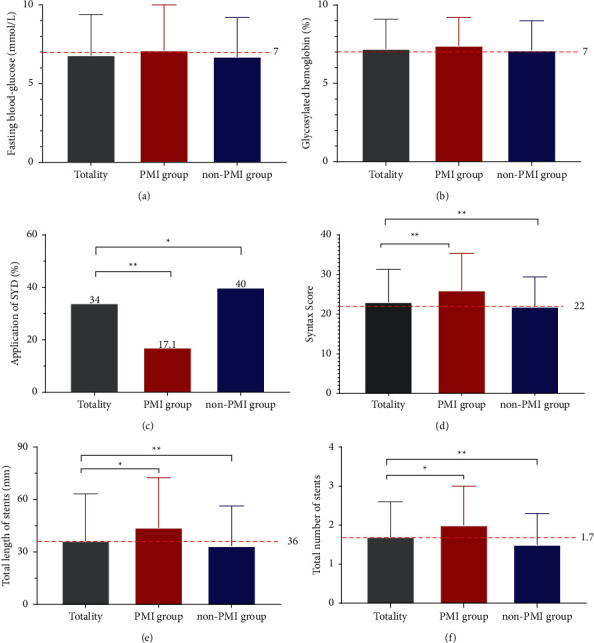
Univariate clinical characteristics associated with PMI. Note: “*∗*” indicates that the difference between the two groups is statistically significant (*P* < 0.05); “∗∗” indicates that the difference between the two groups is statistically different (*P* < 0.01).

Of the 265 investigated patients who undergoing coronary intervention, the mean fasting blood glucose level was nearly 7 mmol/L, the mean glycated haemoglobin level was over 7%, and the mean preoperative SYNTAX score was over 22 points, which is an intermediate-risk category. Comparisons between groups showed that the mean levels of preprocedure SYNTAX score, total number of stents, and total length of stents were higher in the group with PMI than in the group without PMI; the prevalence of SYD, a Chinese medicine prescription with Qi-supplementing and blood stasis-purging, was significantly lower in the group with PMI than in the group without PMI, with a statistically significant difference (*P* < 0.05) (Figure 2).

### 3.2. Characteristics of TCM Clinical Syndromes

**Figure 3:**
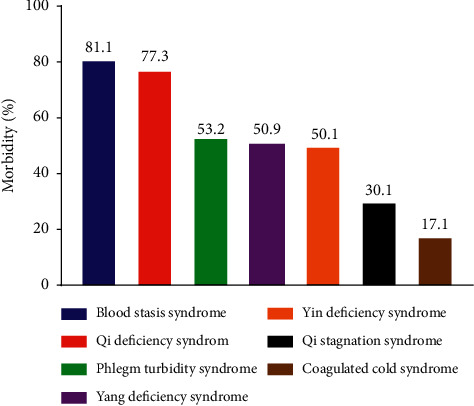
Characteristics of TCM clinical syndromes.

The results of TCM syndromes showed that “Qi deficiency syndrome” and “blood stasis syndrome” were the main TCM syndromes of 265 investigated UA patients who successfully received elective PCI with prevalence rates of 77.3% and 81.1% (Figure 3). The prevalence rates were 50.1% for Yin deficiency syndrome and 50.9% for Yang deficiency syndrome. Phlegm turbidity syndrome, Qi stagnation syndrome, and Coagulated cold syndrome were 53.2%, 30.1%, and 17.1%, respectively.

### 3.3. Factors Associated with PMI Occurrence

**Figure 4:**
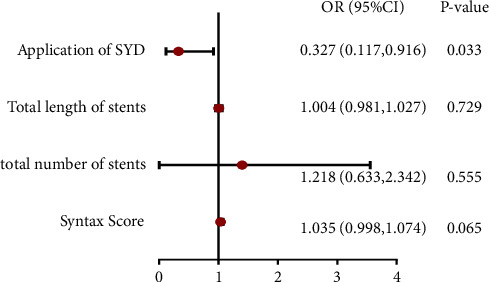
Forest map of factors associated with PMI occurrence.

Using the occurrence of PMI as a dichotomous variable, the four factors that showed statistically significant differences between the groups: preoperative SYNTAX score, number of stents implanted, total length of stents implanted, and the application of SYD were brought into the binary logistic regression equation. The results of the dichotomous logistic regression of potential factors influencing PMI showed that only the application of SYD as a protective factor was statistically significant in the regression equation (OR 0.327, 95% CI 0.117 to 0.916, *P*=0.033) (Figure 4).

## 4. Discussion

This single-center, cross-sectional investigation, case-control study investigated the clinical information of all UA patients who received elective PCI in Beijing Hospital of Traditional Chinese Medicine in the past 2 years. The results showed that nearly one quarter of the UA patients underwent elective PCI had old myocardial infarction; nearly half of the patients had a history of PCI; the prevalence of patients with previous hypertension, type 2 diabetes, and high LDL-C all exceeded 50%; and more than 50% of the patients had triple-vessel disease. The average fasting blood glucose level was nearly 7 mmol/L, the average glycosylated haemoglobin level was over 7%, and the average preoperative SYNTAX score was over 22 points, which was an intermediate-risk category. This showed that patients with UA undergoing elective PCI at this TCM institution had a high level of concomitant disease and coronary artery disease, with a particularly high rate of poor glycemic control and triple-vessel disease in the coronary arteries.

Studies have shown that the incidence of PMI ranges from 4.7 to 40.5% [1, 8–10]. Several factors may be involved in influencing the incidence of PMI in different centers [11, 12]: 1. Pre-PCI coronary condition: compared to stable angina, acute coronary syndrome (ACS) as an acute coronary event caused by unstable plaque rupture may have a higher risk of PMI occurrence. 2. Underlying disease: the more patients have underlying diseases such as hypertension, diabetes mellitus, and high LDL-C that contribute to the aggravation of coronary artery disease, the higher the risk of PMI may be. 3. The technical level of PCI operation in each center: PMI is the myocardial injury caused during PCI operation, and the technical level of the operator will affect the risk of PMI after PCI. 4. Application of drugs with alleviating PMI: studies have shown that the application of a loading dose of atorvastatin calcium before PCI can significantly reduce the occurrence of PMI, but this treatment regimen has not been confirmed in China. 5. Means of detection of PMI: the latest joint ESC/ACCF/AHA/WHF “Universal Definition of Myocardial Infarction, Fourth Edition” [3] issued in 2018 places the preprocedure troponin (cTn) level of PCI. However, studies of PMI in the last 5 years have reported that the diagnostic criteria for PMI vary, for example, elevated CK-MB levels after PCI or elevated cTn above 3 times the upper limit of normal after PCI. These factors may explain, to some extent, the wide variation in the incidence of PMI shown in the current reports. The present study showed that the incidence of PMI after PCI in our center was 26.4%. The clinical characteristics of UA treated with PCI in our center were characterised by a high degree of concomitant disease and coronary lesions, with a particularly high rate of poor glycemic control and coronary triple-vessel disease. Studies have shown that coronary microcirculatory lesions induced during coronary interventions may be an important factor in the development of PMI. In the Chinese Expert Consensus on the Diagnosis and Treatment of Coronary Microvascular Diseases, which was jointly promulgated by several academic teams including the Basic Research Group of the Chinese Society of Cardiovascular Diseases, it was pointed out [13] that atherosclerotic risk factors such as diabetes mellitus, hypertension, and high LDL-C level can be influenced by endothelium-dependent and non-endothelium-dependent mechanisms leading to abnormal microvascular function, manifested by reduced coronary flow reserve fraction and microvascular constriction. Therefore, based on the co-effect of multiple risk factors on the occurrence of PMI, the bad clinical characteristics of patients in our center including a high prevalence of concomitant diseases (more than 50% of the patients with hypertension, type 2 diabetes, and high LDL-C level) and a high degree of coronary lesions (more than 50% of the patients with three lesions) are supposed to be important in explaining the level of PMI in our center.

Clinical evidence of TCM characteristics. “Qi deficiency syndrome” and “blood stasis syndrome” were the main TCM symptoms in the patients investigated in our center, followed by Phlegm turbidity syndrome, Yang deficiency syndrome, YingXu, Qi stagnation syndrome, and coagulated cold syndrome. The results of the study suggest that Qi deficiency syndrome and blood stasis syndrome are the main TCM symptoms in patients undergoing elective PCI and that the method of Qi-supplementing and blood circulation-activating (blood stasis-purging) should be the core treatment method to improve the TCM symptoms of patients. This is similar to what is stated in the expert consensus on the Chinese medicine treatment of perioperative myocardial injury during percutaneous coronary intervention (PCI) [14].

SYD is a TCM prescription with the effect of “Qi-supplementing and blood stasis-purging” based on the pathogenesis characteristics of UA “Qi deficiency and blood stasis syndrome,” which is composed of *Astragalus membranaceus*, *Salvia miltiorrhiza*, *Codonopsis pilosula*, *Scrophularia ningpoensis*, *Corydalis ambigua*, *Hirudo nipponica*, *Eupolyphaga sinensis*, and *Pberetima* (supplementary document). In an experimental study using Chinese mini-pigs as an animal model, SYD was shown to exert myocardial protective effects in the perioperative period of PCI through the inhibition of nuclear factor NF-*κ*B and its mediated oxidative stress and inflammatory response [15], which was more effective in the high dosage group. Randomized, controlled trials suggest that application of SYD at 3 days before PCI can reduce PMI with a good safety profile [16, 17]. However, whether these findings can be extrapolated to the real world needs to be further evaluated. In order to further assess the effect of SYD with Qi-supplementing and blood stasis-purging on the occurrence of PMI under multiple factors, four factors that showed statistically significant differences between groups were brought into the binary logistic regression equation: preoperative SYNTAX score, number of stents implanted, total length of stents implanted, and the application of SYD. The results showed that only the application of SYD as a protective factor was statistically significant in the regression equation, suggesting that the reduction in the occurrence of PMI may be due to the application of SYD.

Study limitations. 1. Limitations in sample size and study center. This study applied a 2-year period to collect 265 UA patients in a single center, which initially provided some data information on the incidence of PMI in Chinese medical institutions and the clinical characteristics of patients. However, the result in this study showed that the incidence of PMI was 26.4%, which was lower than the assumed that of 30% when estimating the sample size, which led to an underestimation of the sample size. As a result, the limited sample size and study center may have limited further mining of relevant data. For example: in the forest plot of the multiple regression model, the four factors had significant gaps in the confidence intervals of the regression equation, and the high level of SYNTAX score as a potential risk factor had a proximate statistical difference in the regression analysis (*P*=0.065), both of which may be related to the limited sample size of this study. In addition, the limited sample size is also supposed to affect the further exploration of the co-effect of multiple risk factors and the influence of other frequently used TCM drugs as potentially protective factors on the disease. Nevertheless, it is with value that this study can provide a reference for the further exploration above. 2. Limitations of the study type. The present study is a retrospective study, which gives us a hint that the reduction in PMI may be due to the application of the SYD by “deducing the cause from the effect”, but we cannot draw a conclusion that SYD is useful in reducing the occurrence of PMI. Further clinical trials, including prospective cohort studies, will be required if we want to acquire a clear evidence of this causal relationship in the real world.

Study prospect. 1. To enhance the exploration of the association between the occurrence of PMI and distant outcome events. This study showed the incidence of PMI in our center but does a higher level of PMI predict the occurrence of high-risk adverse outcome events in patients? Can this potential high risk of adverse outcome events be improved by the use of Chinese medicine, especially the application of SYD? Further studies are needed. 2. Further comparisons with Western medical institutions. The group has previously conducted a 10-year registration survey on the treatment of patients with AMI in Beijing's tertiary care Chinese medicine hospitals and has investigated eight tertiary care Western medicine hospitals in Beijing as controls, with a cumulative total of nearly 5,000 cases. The results showed that AMI patients in TCM hospitals had independent clinical characteristics, including late arrival at hospital, high age of onset, high proportion of women, more past medical history, more accompanying diseases, and more complications [2, 18]. This separate clinical profile made the demographic baseline information of AMI patients in Chinese medicine hospitals significantly different from that in Western medicine hospitals, and therefore, the treatment status and prognosis of the two differ. In general randomized controlled studies, patients with severe conditions such as “advanced age” and “severe hepatic and renal insufficiency” are often excluded from clinical evaluation as exclusion criteria. However, this part of the population may be the majority of people in TCM hospitals. UA has the same pathophysiological mechanisms as AMI, so a study of the clinical characteristics of patients undergoing PCI in Western medical institutions based on the present study may be useful in the development of further targeted treatment protocols for patients attending TCM hospitals.

## 5. Conclusion

Patients with UA who received elective PCI in TCM institutions may have clinical characteristics including multiple accompanying diseases and high stenosis coronary artery, in which the incidence of poor blood glucose control and high rate of three-vessel coronary disease are particularly significant. The TCM syndromes are mainly Qi deficiency and blood stasis syndromes. The decrease of PMI may be attributed to the application of SYD in the real world, which however needs to be further evaluated by prospective cohort studies.

## Figures and Tables

**Table 1:** Clinical data and comparative analysis according to the occurrence of PMI.

Variable	Totality *n* = 265	PMI group, *n* = 70 (26.4%)	Non-PMI group, *n* = 195 (73.6%)	*χ* ^2^ (F)	*P* value
Male patients	162 (61.1%)	46 (65.7%)	116 (59.5%)	0.841	0.657
Age	63.5 ± 9.7	62.2 ± 9.8	63.9 ± 9.6	0.836	0.434
Smoking	138 (52.1%)	36 (51.4%)	104 (53.3%)	0.105	0.949
Drinking	86 (32.5%)	22 (31.4%)	64 (32.8%)	0.046	0.977
Old myocardial infarction	62 (23.4%)	16 (22.9%)	46 (23.6%)	0.015	0.992
History of PCI	120 (45.3%)	32 (45.7%)	88 (45.1%)	0.007	0.996
History of CABG	1 (0.4%)	1 (1.4%)	0	2.673	0.263
Hypertension	201 (75.8%)	52 (74.3%)	149 (76.4%)	0.127	0.939
Type 2 diabetes	151 (57.0%)	42 (60.0%)	109 (55.9%)	0.354	0.838
High LDL-C	176 (69.3%)	54 (77.1%)	133 (68.2%)	2.057	0.357
Old cerebral infarction	71 (26.8%)	15 (21.4%)	56 (28.7%)	1.395	0.498
Statins	234 (88.2%)	63 (89.4%)	171 (87.7%)	0.122	0.941
beta-blockers	161 (60.6%)	44 (62.1%)	117 (59.9%)	0.098	0.952
ACEI/ARB	117 (44.2%)	32 (46.2%)	85 (43.4%)	0.138	0.933
Fasting blood glucose (mmol/L)	6.8 ± 2.6	7.1 ± 2.9	6.7 ± 2.5	0.833	0.453
Glycosylated haemoglobin (%)	7.2 ± 1.9	7.4 ± 1.8	7.1 ± 1.9	0.836	0.434
Total cholesterol (mmol/L)	4.19 ± 1.18	4.30 ± 1.24	4.15 ± 1.16	0.431	0.65
Low-density cholesterol (mmol/L)	2.41 ± 0.87	2.54 ± 0.99	2.37 ± 0.81	1.007	0.336
Creatinine (umol/L)	70.2 ± 23.6	74.9 ± 24.2	68.5 ± 24.2	1.863	0.156
Grace risk score	102.4 ± 21.1	100.3 ± 20.3	103.2 ± 21.3	0.47	0.626
SYNTAX score^△#^	23.05 ± 8.24	26.03 ± 9.29	21.89 ± 7.51	6.405	0.002
Total number of stents^△#^	1.7 ± 0.9	2.0 ± 1.0	1.5 ± 0.8	5.509	0.004
Mean diameter of stents (mm)	2.70 ± 0.89	2.64 ± 0.78	2.73 ± 0.79	0.312	0.732
Total length of stents (mm)^△#^	36.30 ± 24.94	43.94 ± 28.50	33.38 ± 22.86	4.478	0.012
Application of TCM	247 (93.2%)	64 (91.4%)	183 (93.8%)	0.476	0.788
Application of compound Danshen dripping pill	24 (9.1%)	7 (10%)	17 (8.7%)	0.083	0.469
Application of Tongxinluo capsule	23 (8.7%)	6 (8.6%)	17 (8.7%)	0.001	0.595
Application of Chinese herbal medicine	224 (84.5%)	58 (82.9%)	166 (85.1%)	0.203	0.391
Application of SYD^△#^	90 (34.0%)	12 (17.1%)	78 (40.0%)	11.999	0.002
Application of Danhong injection	153 (57.7%)	36 (51.4%)	117 (60%)	1.551	0.135
Application of Tanshinone IIA injection	93 (35.1%)	27 (38.6%)	66 (33.8%)	0.505	0.285
Triple-vessel disease	133 (50.2%)	38 (54.5%)	95 (48.6%)	0.639	0.727

Note. Variables are expressed as mean ± standard deviation or rate (%); “^△^” indicates a statistically significant difference between the population with PMI and the overall population (*P* < 0.05); “^#^” indicates a statistically significant difference between the population with PMI and the population without PMI (*P* < 0.05).

## Data Availability

The data used to support the findings of this study are available from the corresponding author upon request.

## Abbreviations

ACEI: Angiotensin-converting enzyme inhibitors
ARB: Angiotensin receptor blockers
AMI: Acute myocardial Infarction
CABG: Coronary artery bypass grafting
cTnT: Cardiac troponin T
ECG: Electrocardiograph
EMRs: Electronic medical records
LDL-C: Low-density lipoprotein cholesterol
MACEs: Major adverse cardiovascular events
PCI: Percutaneous coronary intervention
PMI: Periprocedural myocardial injury
PMIf: Periprocedural myocardial infarction
SYD: Shen-Yuan-Dan
TCM: Traditional Chinese medicine
UA: Unstable angina
URL: Upper reference limit.

## Supplementary Materials

Supplementary Materials This table is an overview of SYD, a TCM prescription, which is composed of eight Chinese herbs with a description including Chinese name, Common name, Latin name, and the proportion of each Chinese herb in the formula.
