# Supplementary material for: Clinical Characteristics and Risk Factors of Periprocedural Myocardial Injury in Patients with Elective PCI in a TCM Hospital
Source: Evid Based Complement Alternat Med. 2022 Mar 24;2022:7158740. doi: 10.1155/2022/7158740 (PMC8970869; doi:10.1155/2022/7158740)
Supplement: Supplementary Materials — This table is an overview of SYD, a TCM prescription, which is composed of eight Chinese herbs with a description including Chinese name, Common name, Latin name, and the proportion of each Chinese herb in the formula. [file 7158740.f1.docx]

**Overview of SYD**

| Chinese name | Common name | Latin name | Amount |
| --- | --- | --- | --- |
| Huangqi | Radix astragali | Astragalusmembranaceus | 20.1% |
| Danshen | Root of red-rooted salvia | Salvia miltiorrhiza | 20.1% |
| Dangshen | Root of hairy asiabell | Codonopsispilosula | 20.1% |
| Xuanshen | Figwort | Scrophularianingpoensis | 20.1% |
| Yanhusuo | Rhizomacorydalis | Corydalis ambiguaCh | 6.7% |
| Shuizhi | Leech | Hirudonipponica | 2.1% |
| Tubie | Ground beetle | Eupolyphagasinensis | 4.1% |
| Dilong | Earthworm | Pberetima | 6.7% |

Supplementary description: This table is an overview of SYD, a TCM prescription, which is composed of eight Chinese herbs with a description including *Chinese name, Common name, Latin name,* and *the proportion of each Chinese herb in the formula.*
